# Genetic alterations in Thai adult patients with acute myeloid leukemia and myelodysplastic syndrome—excess blasts detected by next-generation sequencing technique

**DOI:** 10.1007/s00277-021-04513-z

**Published:** 2021-04-10

**Authors:** Weerapat Owattanapanich, Julia Herzig, Nikolaus Jahn, Ekaterina Panina, Theera Ruchutrakool, Smith Kungwankiattichai, Surapol Issaragrisil, Hartmut Döhner, Konstanze Döhner

**Affiliations:** 1 Department of Internal Medicine III, University Hospital of Ulm, 89081 Ulm, Germany; 2 Division of Hematology, Department of Medicine, Faculty of Medicine Siriraj Hospital, Mahidol University, Bangkok, 10700 Thailand

**Keywords:** Acute myeloid leukemia, Genetic, Molecular, Next-generation sequencing, Thailand

## Abstract

Several molecular aberrations affect the prognosis of patients with acute myeloid leukemia (AML) and myelodysplastic syndrome (MDS) with excess blasts (EB). This study aimed to determine the incidence and clinical impact of molecular genetic aberrations in Thai patients with AML and MDS-EB, detected by the next-generation sequencing (NGS) technique. This prospective, observational study was conducted between 2018 and 2020 on newly diagnosed Thai AML or MDS-EB patients aged above 15 years. NGS was performed using a custom amplicon-based targeted enrichment assay for 42 genes recurrently mutated in myeloid neoplasms. The molecular results were correlated with baseline patient and disease characteristics as well as outcomes. Forty-nine patients were enrolled in this study. The median age was 56 years (interquartile range [IQR], 44–64), with nearly equal proportions of males and females. The median number of mutations was 3 (IQR, 2–4). The most frequent alterations were *FLT3* internal tandem duplications (ITD) (28.6%), *DNMT3A* (24.5%), and *WT1* (22.4%) mutations. *FLT3*-ITD was more frequent in the de novo AML group than in the MDS/secondary AML group, whereas in the MDS/secondary AML group, *ASXL1*, *ETV6,* and *SRSF2* mutations were more frequent. Patients aged greater than 65 years and patients with mutated *TP53* were more likely to have inferior overall survival from multivariate analysis. *FLT3*-ITD was the most common mutation among newly diagnosed Thai AML patients. *TP53* mutation and advanced age were independent adverse factors for survival outcome. The genetic landscapes of AML patients vary between national populations. Thai Clinical Trials Registry identifier: TCTR20190227003.

**Supplementary Information:**

The online version contains supplementary material available at 10.1007/s00277-021-04513-z.

## Introduction

Currently, two main standard recommendations for acute myeloid leukemia (AML), published by the European LeukemiaNet (ELN) and the National Comprehensive Cancer Network, are being used to determine patients’ risk stratification, and to select the appropriate therapy [1, 2]. Stratification of risk at the time of diagnosis is primarily based on cytogenetic and molecular genetic findings. Results from the genetic workup are also essential for determining and guiding an appropriate, long-term, treatment strategy. For instance, patients with AML harboring *NPM1* mutation and *FLT3* wildtype or *FLT3*-ITD with low allelic ratio, AML with biallelic *CEBPA* mutation, or with core-binding factor AML are categorized within the favorable risk group and may require only induction chemotherapy followed by consolidation chemotherapy [1, 2]. In contrast, allogeneic stem cell transplantation after induction therapy is generally recommended for patients with adverse genetic profiles, such as complex karyotype or mutated *RUNX1*, *ASXL1*, or *TP53* [1–6].

In addition, personalized treatment is widely integrated with AML treatment strategies, depending on mutational status; for example, the *FLT3* inhibitor midostaurin combined with intensive induction and consolidation chemotherapy followed by a 1-year maintenance therapy yielded significantly better survival outcomes of patients with *FLT3*-mutated AML [7, 8]. The IDH1 inhibitor ivosidenib and IDH2 inhibitor enasidenib show encouraging clinical activity in patients with *IDH*-mutated AML [9, 10]. Furthermore, myelodysplastic syndrome (MDS) with excess blasts-2 (EB2) shares a similar natural history with AML [11]. Therefore, a comprehensive genetic investigation of MDS-EB patients is required, just as for patients with AML.

Reverse transcriptase-polymerase chain reaction (RT-PCR) is a conventional genetic testing method that can be performed by the majority of institutes. Nevertheless, the main limitation of this technique is that each mutation has to be evaluated separately, which is time-consuming. This is in particular clinically relevant for patients with newly diagnosed AML and MDS-EB. Over the last years, the next-generation sequencing (NGS) technique was introduced for myeloid neoplasms [12, 13]. The technique can be used to evaluate several target genes within a few days. Health-care institutes, especially those in developed countries, are increasingly adopting the NGS method to investigate the mutational status of newly diagnosed AML and MDS-EB patients. However, comprehensive genetic profiles for the Southeast Asian population, including Thais, have not been fully studied.

We, therefore, performed this study primarily to determine the incidence of molecular aberrations in Thai patients with AML and MDS-EB, as detected by the NGS technique. The secondary objective was to correlate molecular mutational status with clinical outcomes and to evaluate the genetic landscape differences between Thais and other ethnic groups of AML and MDS patients.

## Methods

This prospective observational study was conducted on newly diagnosed Thai AML and MDS-EB patients between January 2018 and March 2020. The inclusion criteria were as follows: (1) patients aged above 15 years; (2) patients with de novo AML, secondary AML, or MDS-EB; and (3) patients requiring treatment and follow-up at Siriraj Hospital, Thailand. Every participant signed a consent form before enrollment. The mononuclear cells from bone marrow specimens were collected and cryopreserved in a biobank. The genomic deoxyribonucleic acid (gDNA) was isolated using the QIAGEN Genomic DNA extraction kit (Qiagen, Hilden, Germany). The qualities and concentrations of gDNA were confirmed by gel electrophoresis and a Qubit 3.0 Fluorometer (life technologies by Thermo Fisher Scientific, Waltham, MA, USA). All of the gDNA products were transferred to the University Hospital of Ulm, Germany, for the performance of the molecular study. The Siriraj Institutional Review Board approved this research, which followed the Declaration of Helsinki guidelines and all subsequent amendments. The study was approved for registration at the Thai Clinical Trial Registry and the identification number is TCTR20190227003. Molecular studies were supported by the Department of Internal Medicine III, University Hospital of Ulm, Ulm, Germany.

### NGS library preparation and data interpretation

The NGS covered entire coding regions of 42 genes recurrently mutated in myeloid neoplasms by using a custom amplicon-based targeted enrichment assay (HaloplexHS Target Enrichment System, Agilent, Santa Clara, CA, USA). The library preparation was performed according to the manufactures’ instructions. The library products were sequenced on a MiSeq sequencer using the 300-cycle MiSeq Reagent Kit v2 (Illumina, San Diego, CA, USA). Following demultiplexing, the paired-end sequences were analyzed by an in-house data analysis workflow. In brief, sequences were aligned to the human reference genome GRCh37 (hg19) using BWA-MEM (version 0.7.10) [14]. Based on the molecular barcodes, duplicates were removed and consensus sequences were generated (BamDeduplicateByBarcode, ngs-bits). After local realignment by GATK (version 3.4-16) [15], variants were called using VarScan (v2.3.9) [16] and annotated by ANNOVAR [17]. Semi-automated filtering was applied with intronic variants, synonymous variants, variants with less than one tumor supporting read, and variants with an entry in the Database of Single Nucleotide Polymorphisms (dbSNP, build 138) [18], but not in the Catalogue of Somatic Mutations in Cancer (COSMIC) [19] that were filtered out. The heterozygous variant allele frequency (VAF) detection threshold was 3%. The remaining variants were manually analyzed and curated by the Viewer Integrative Genomics (IGV, California, USA), the UCSC Genome Browser, the COSMIC database, and dbSNP [20, 21]. *NPM1* and *FLT3*-ITD were determined by DNA-based assays, *FLT3*-ITD as described by Stone et al. [7].

### Terminology

This study applied the 2017 ELN risk classification based on the cytogenetic and molecular findings [1]. Secondary AML in this cohort was defined as the patient with a previous history of MDS. CR was defined as < 5% blasts in the bone marrow, an absolute neutrophil count of ≥ 1.0 × 10^9^/l, and a platelet count of ≥ 100 × 10^9^/l, without any of the following: (1) circulating blasts, (2) blasts with Auer rods, and (3) extramedullary disease. The OS was defined as the duration from diagnosis to the time of last follow-up or death, whereas the RFS was defined as the duration from the CR date to the date of relapse or death from any cause.

### Statistical analysis

Continuous data were reported as median with interquartile range (IQR) or mean ± standard deviation, as suitable. The Mann–Whitney *U* test or Student’s *t*-test was employed to compare continuous data. Categorical data were presented as number and percent, and compared using Fisher’s exact test or a chi-square test. A log-rank test was used to compare the factors correlated with OS and RFS, and it was presented as a Kaplan–Meier survival curve. Cox proportional hazards analysis (enter method) was used to compare the predictors of survival outcome in the univariate and multivariate analyses. The independent variables that have significance in univariate analysis entered the multivariate model. The results were expressed as hazard ratio (HR) and 95% CI. Statistical significance was determined as a *p*-value of < 0.05. The program IBM SPSS Statistics for Windows, version 20.0 (Armonk, NY: IBM Corp.), was utilized to analyze the data.

## Results

Forty-nine cases were enrolled in this study. The median age was 56 years (IQR, 44–64), with almost equal proportions of males and females. Forty-six (93.9%) patients had AML in which 39 patients (79.6%) were de novo and 7 patients (14.3%) were secondary AML. Two acute promyelocytic leukemia (APL) patients were found in these AML patients. The others were MDS-EB, accounting for 3 (6.1%) patients. The common initial clinical manifestations included anemia symptoms (87.8%), fever (40.8%), bleeding (28.6%), and significant weight loss (24.5%). Table 1 summarizes the baseline patient, disease characteristics, and cytogenetic risks.

**Table 1:** Baseline clinical and disease characteristics of the 49 patients

	Number (%)
Age (years), median (IQR)	56 (44-64)
Male sex, *n* (%)	24 (49)
Disease types, *n* (%)	
*- De novo* AML	39 (79.6)
- Secondary AML	7 (14.3)
- MDS-EB	3 (6.1)
Laboratory findings	
Complete blood count	
- Mean Hb level (g/dl)	7.5 ± 2.5
- Median WBC count (×10^9^/l)	28.3 (IQR, 10.7–85.5)
- Median peripheral blood blasts (%)	59 (IQR, 26–82)
- Median platelet count (×10^9^/l)	45 (IQR, 20–67)
Median bone marrow blasts (%)	72.5 (IQR, 47.5–90.0)
AML cytogenetic risk according to 2017 ELN risk classification	
- Favorable risk	5 (10.2)
- Intermediate risk	32 (65.3)
- Adverse risk	7 (14.3)
- No result and MDS cases	5 (10.2)
Number of molecular mutations	
- No mutations	1 (2.0)
- 1 mutation	5 (10.2)
- 2 mutations	10 (20.4)
- 3 mutations	12 (24.5)
- 4 mutations	10 (20.4)
- 5 mutations	5 (10.2)
- 6 mutations	3 (6.1)
-7 mutations	2 (4.1)
- 8 mutations	1 (2.0)

*AML*, acute myeloid leukemia; *EB*, excess blasts; *Hb,* hemoglobin; *IQR*, interquartile range; *MDS*, myelodysplastic syndrome; *WBC,* white blood cell

### Molecular landscape results

**Fig. 1:**
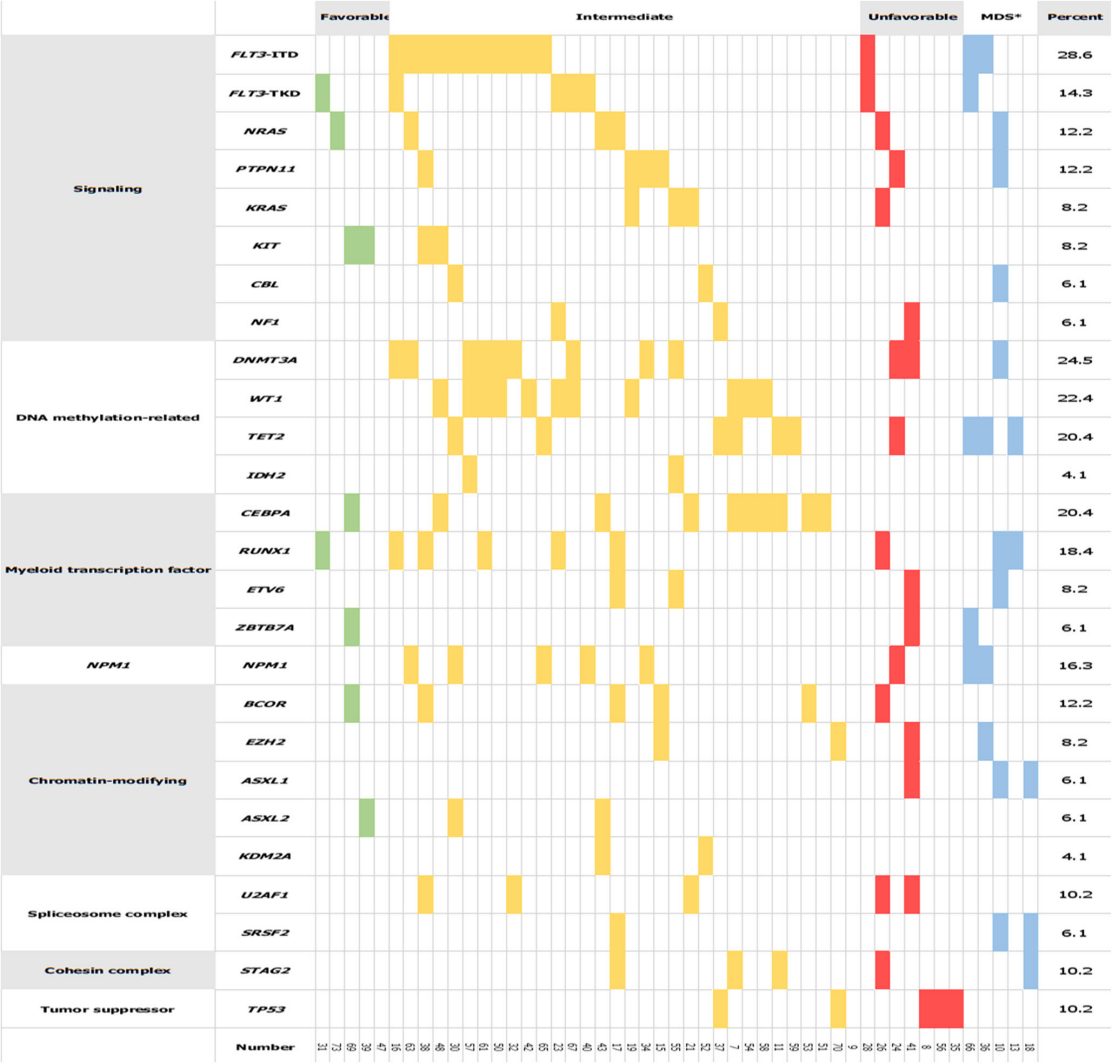
Oncoplot for the 49 patients with molecular mutations

The median number of gene mutations was 3 (IQR, 2–4). The most common mutations were *FLT3*-ITD (28.6%, as assessed by conventional diagnostic assay), *DNMT3A* (24.5%), *WT1* (22.4%), *TET2* (20.4%), *RUNX1* (18.4%), *NPM1* (16.3%), *FLT3*-TKD (14.3%), monoallelic *CEBPA* (10.2%), and biallelic *CEBPA* (10.2%). The favorable molecular genotypes *NPM1* mutation without *FLT3*-ITD or with *FLT3-*ITD^low^ and the biallelic *CEBPA* mutation represented 6.1% and 10.2% of cases, respectively. The targeted gene sequencing results of the 49 cases, classified by functional gene, [1] are illustrated in Fig. 1.

**Fig. 2:**
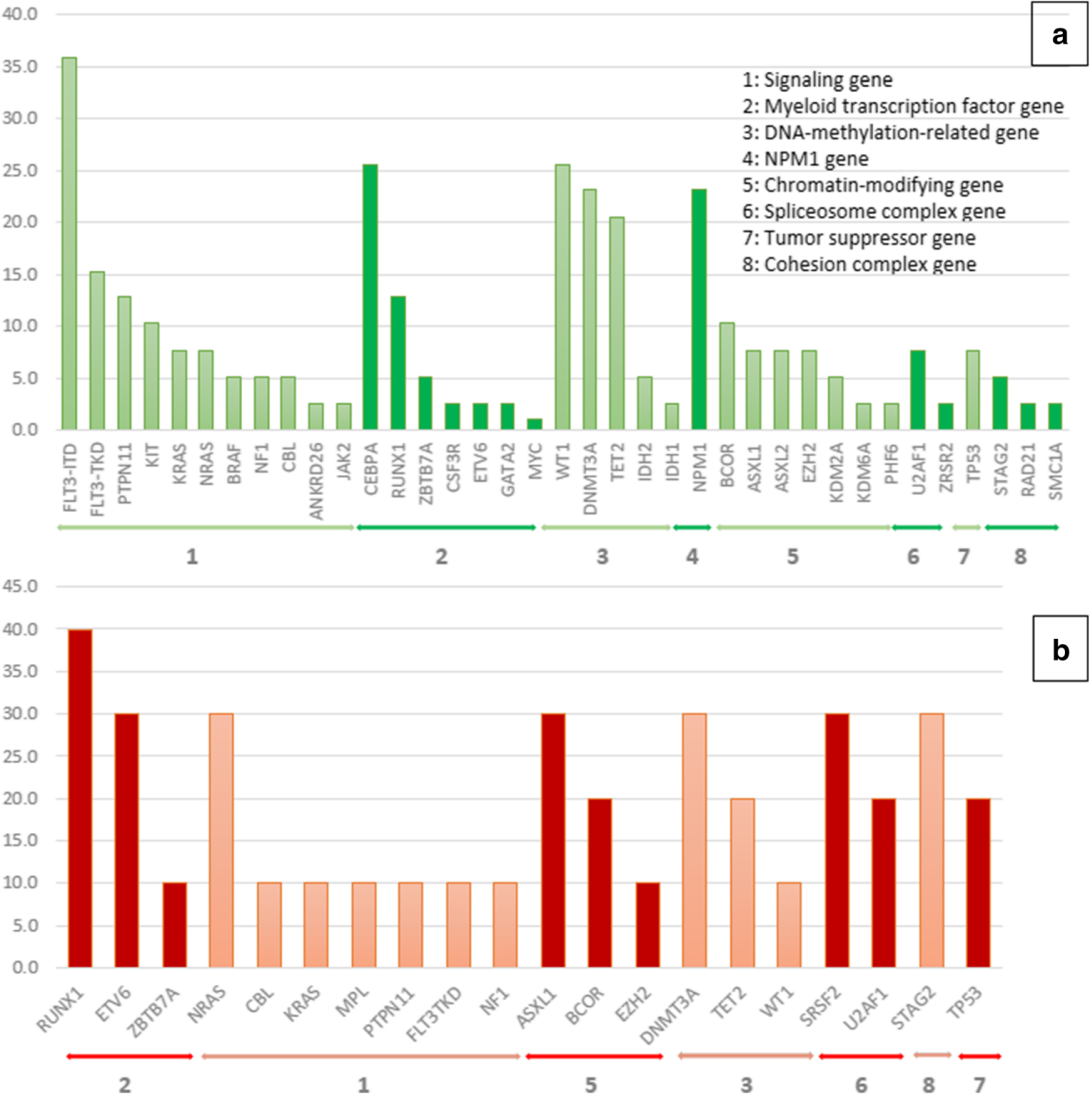
Frequencies of each genetic alteration of the Thai patients, categorized by disease type: (A) *De novo* AML group; (B) MDS/secondary AML

Because the genetic mutations and pathogenesis of MDS and secondary AML are very similar [11], we divided the patients into two groups: a de novo AML group (*n*=39), and an MDS combined with a secondary AML group (*n*=10). The common genetic alterations of the de novo group were *FLT3*-ITD (35.9%), monoallelic or biallelic *CEBPA* (25.6%), *WT1* (25.6%), *DNMT3A* (23.1%), *NPM1* (20.5%), *TET2* (20.5%), and *FLT3*-TKD (15.4%), while the common ones in the latter group were *RUNX1* (40.0%), *ASXL1* (30.0%), *DNMT3A* (30.0%), *ETV6* (30.0%), *NRAS* (30.0%), *STAG2* (30.0%), *SRSF2* (30.0%), *BCOR* (20.0%), *TET2* (20.0%), *TP53* (20.0%), and *U2AF1* (20.0%). Figure 2 displays the frequencies of the genetic alterations found in the Thai patients categorized by disease type. As to the baseline patient characteristics of the two groups, significantly higher white blood cell counts and higher percentages of peripheral blood blasts and marrow blasts were detected in the de novo AML group than in the MDS/secondary AML group. A comparison of the genetic profiles of the two groups revealed that the *FLT3*-ITD mutation was more likely to be present in the de novo AML group than in the MDS/secondary AML group (*p* = 0.044). In contrast, the MDS/secondary AML group more frequently had mutations in *ASXL1*, *ETV6*, and *SRSF2* genes (*p*=0.007, 0.023, and 0.007, respectively: Table S1).

**Fig. 3:**
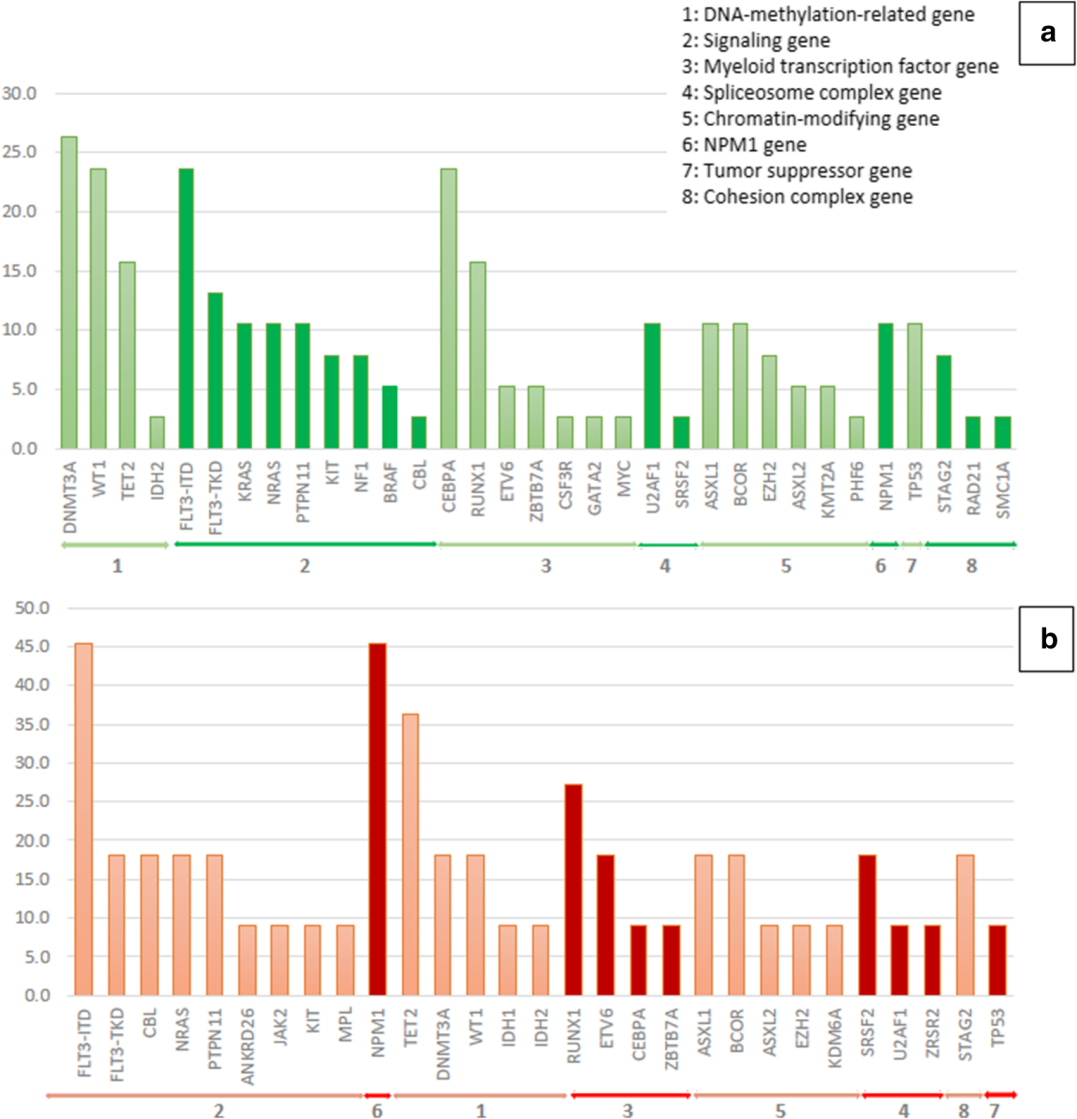
Frequencies of each genetic alteration of the Thai patients, categorized by age group: (A) aged ≥ 65 years; (B) aged < 65 years

Furthermore, we compared the clinical features and mutational status of two age groups, < 65 years (*n*=38) and ≥ 65 years (*n*=11). The elderly group had a significantly greater proportion of patients with a poor Eastern Cooperative Oncology Group performance status. The common mutations in the younger group were *DNMT3A* (26.3%), monoallelic or biallelic *CEBPA* (23.7%), *FLT3*-ITD (23.7%), *WT1* (23.7%), *RUNX1* (15.8%), and *TET2* (15.8%). In the elderly group, the common mutations were *FLT3*-ITD (45.5%), *NPM1* (36.4%), *TET2* (36.4%), and *RUNX1* (27.3%). The proportions of all molecular aberrations found in the two age groups of the Thai patients are illustrated in Fig. 3. There was no significant difference in the proportion of all mutations between the two age groups (Table S2).

### Treatment response and clinical outcomes

Sixty-one percent of patients received intensive induction therapy (7+3 regimen, *n* = 29; 5+2 regimen, *n* = 1), 10.2% hypomethylating agents (HMAs), and 4.1% APL induction. The CR rate of the patients who received intensive induction therapy reached 60%, while the induction mortality rate was 11.4%. A quarter (24.5%) of the patients received supportive treatment or low-intensive therapy, comprised of hydroxyurea, oral low-dose chemotherapy, and subcutaneous cytarabine. An allogeneic stem cell transplant after CR using a matched sibling donor was performed in only one patient because of the cost-effectiveness-based transplantation policy in Thailand, under which only matched-sibling donor allogeneic stem cell transplants are approved. There was no difference in the CR rates of the de novo AML and the MDS/secondary AML groups (63.3% and 66.7%, respectively).

**Fig. 4:**
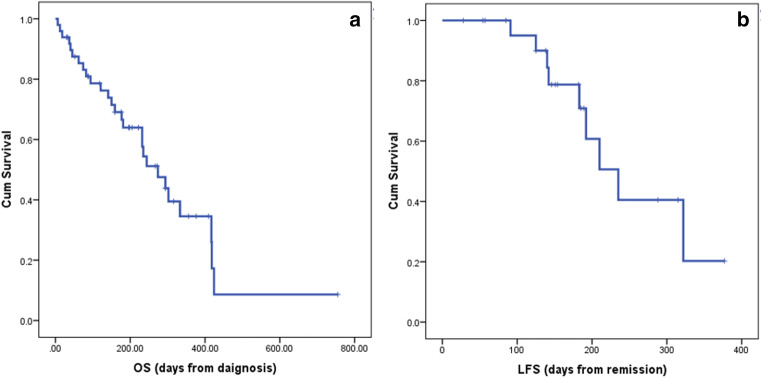
Kaplan–Meier survival curves for all patients: (**a**) overall survival; (**b**) relapse-free survival

**Fig. 5:**
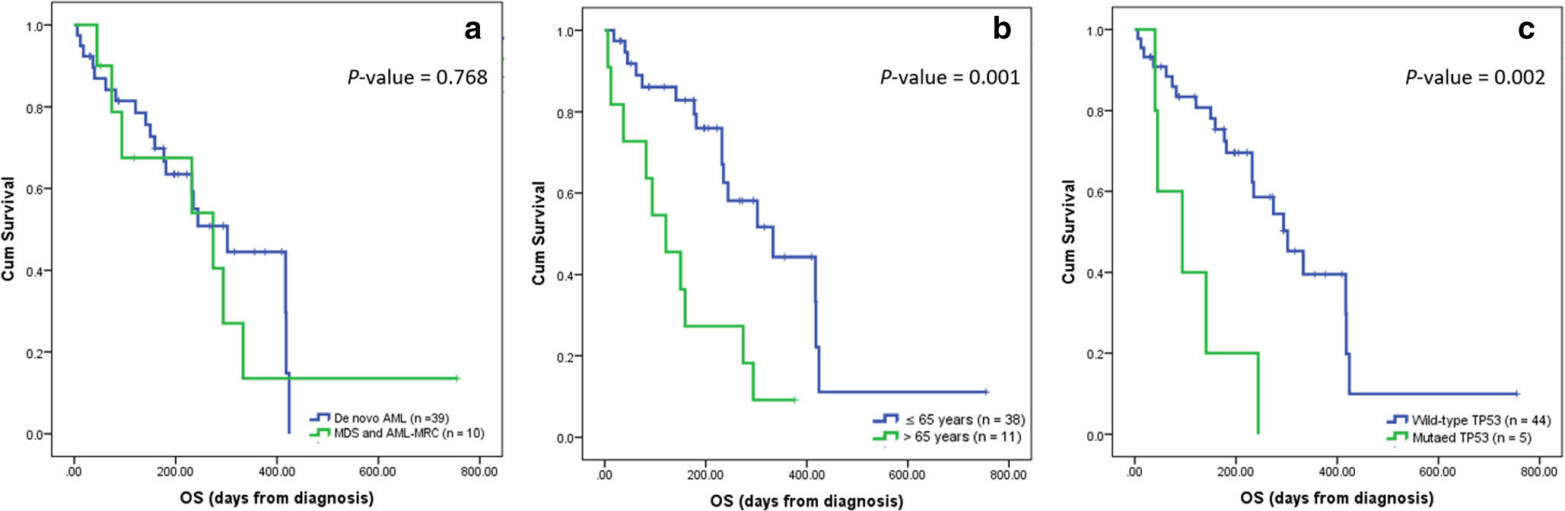
Kaplan–Meier survival curves for the patients, classified by disease type, age group, and genetic mutation: (**a**) disease type; (**b**) age group; (**c**) *TP53* mutational status

The median OS time of all patients was 274 days (95% confidence interval [CI]: 199.83–348.17), with a 1-year OS rate of 33.8%; the median RFS time was 235 days (95% CI: 173.59–296.41), with a 1-year RFS rate of only 24.9%. The Kaplan–Meier survival curves for the OS and RFS outcomes of the entire cohort are illustrated in Fig. 4. A subgroup analysis of several factors impacting on survival outcome did not identify any difference in the OS outcomes of the de novo AML group and the MDS/secondary AML group (the median OS times were 303.6 days and 273.4 days, respectively; hazard ratio [HR]: 1.19; 95% CI: 0.50–2.87; *p* = 0.687). The patients who were aged 65 years or less had a significantly better OS than those aged over 65, with a median OS time of 355.9 days versus 135.0 days, respectively (HR: 3.51; 95% CI: 1.55–7.94; *p* = 0.003). The former group received intensive chemotherapy accounting for 89.5% compared to only 9.1% in the latter group. As to the mutational status analysis, the *TP53* was the only mutation that showed a statistically significant difference in survival outcomes. Patients with wild-type *TP53* had a median OS time of 300.4 days, whereas those with mutated *TP53* had a median OS time of 105.0 days (HR: 4.45; 95% CI: 1.59–12.46; *p* = 0.004). Figure 5 presents the Kaplan–Meier survival curves of the patients, classified by disease type, age group, and genetic mutation*.* A multivariate analysis was performed to assess all possible factors that were significantly correlated to the survival time of the patients. Patients aged greater than 65 years and patients with the mutated *TP53* were more likely to have an inferior OS, with the HRs of 3.22 (*p* = 0.006) and 4.38 (*p* = 0.006), respectively (Table 2).

**Table 2:** Univariate and multivariate analyses for overall survival of the patients (days)

Univariate	N	Overall survival (days)	*P*-value	HR (95 % CI)
Age			***0.003***	
≤ 65 years	38	335.9		1
> 65 years	11	135.0		3.51 (1.55–7.94)
Disease types			0.687	
*De novo* AML	39	303.6		1
MDS combined with AML	10	273.4		1.19 (0.50–2.87)
Cytogenetic risk			0.244	
Favorable risk	5	310.0		1
Intermediate and adverse risk	39	260.1		3.40 (0.43-26.63)
*TP53* mutational status			***0.004***	
Wild-type *TP53*	44	300.4		1
Mutated *TP53*	5	105.0		4.45 (1.59–12.46)
*CEBPA* mutational status			0.103	
Non-biallelic *CEBPA* gene mutation	44	236.2		1
Biallelic *CEBPA* gene mutation	5	412.5		1.19 (0.03–1.40)
Multivariate	*P*-value	HR (95 % CI)		
Age	*0.006*	3.22 (1.39–7.40)		
*TP53* mutational status	*0.006*	4.38 (1.53–12.51)		

A *p*-value of < 0.05 indicates statistical significance

*AML*, acute myeloid leukemia; *CI,* confidence interval; *HR,* hazard ratio; *MDS*, myelodysplastic syndrome

## Discussion

This is the first prospective study to comprehensively investigate the genetic profiles of Thai patients with AML and MDS-EB using the NGS technique. *FLT3*-ITD (assessed by conventional PCR technique) was the most common genetic mutation in this cohort. Other mutations that were commonly found in our study were mutations in DNA methylation (*DNMT3A* and *TET2*), the myeloid transcription factor (*CEBPA* and *RUNX1*), and the *NPM1* gene, known to be frequently mutated in AML. However, the high proportion of *WT1* mutation in our results was markedly different from the frequencies reported by other studies [22–25].

In de novo AML patients, the most common genetic mutations were *FLT3*-ITD, *NPM1,* and *CEBPA*, which is in line with another report from an Asian cohort [26]. *DNMT3A* mutation was commonly found in a high percentage especially in younger AML patients, which compares well with a previous report [27].

As to the MDS/secondary AML patients, mutations in the myeloid transcription factor (*RUNX1* and *ETV6*), chromatin-modifying gene (*ASXL1* and *BCOR*), spliceosome complex (*SRSF2* and *U2AF1*), cohesion complex gene (*STAG2*), and tumor suppressor gene (*TP53*) were demonstrated in high proportions compared with the frequencies in the de novo AML patients; this finding was similar to the results of previous reports. As previously shown, these mutational groups appear to be a signature of this patient subgroup [1, 12, 28, 29].

Notably, just over 10% of the patients aged 65 years or less had mutated *NPM1,* whereas the rate in a previous report was as high as 30% [1]. Besides, high percentages of unfavorable mutations were found in elderly patients, including *FLT3*-ITD and *RUNX1* mutations. These genetic profiles are possibly important adverse factors for Thai elderly patients with AML, and the profiles appear to support the published results of a study of Thai elderly patients with AML who had dismal survival outcomes, with a median OS time of only 4 months [30]. However, the percentages of mutations in the elderly group need to be interpreted with caution due to the low number of patients. The frequencies of the genetic mutations detected by the NGS technique in the AML patients in the present study and previous research are tabulated in Table 3 [4, 22–24, 31].

**Table 3:** Proportions of mutations in AML patients in this study and other research

	Lin 2017 [22]	Hussaini 2018 [23]	Cao 2018 [24]	Eisfeld 2018 [31]	This study
Patients’ country	Taiwan	USA	China	USA	Thailand
Patient numbers	112	187	179	423	49
Gender (M/F)	67/45	NR	116/63	251/172	24/25
Median age **(**years, range)	42.6 (11.7–79)	NR	53 (18–88)	69 (60–85)	56 (15–89)
*FLT3*-ITD mutation	**21.4**	11	10.1	20.9	**28.6**
*FLT3*-TKD mutation	NR	NR	2.8	6.7	14.3
*NRAS* mutation	NR	11.9	11.7	11.6	12.2
*PTPN11* mutation	NR	NR	NR	4.9	12.2
*KRAS* mutation	NR	0.5	2.2	2.4	8.2
*KIT* mutation	4.5	2.1	7.8	NR	8.2
*CBL* mutation	NR	1.1	NR	2.4	6.1
*NF1* mutation	NR	0.5	NR	NR	6.1
*DNMT3A* mutation	12.5	14.8	8.9	**26.9**	**24.5**
*WT1* mutation	11.6	NR	5.0	3.8	**22.4**
*TET2* mutation	10.7	**15.3**	**16.8**	**26.7**	20.4
*IDH2* mutation	12.5	12	10.1	18.4	4.1
*IDH1* mutation	3.6	6.4	6.7	10.4	2.0
*CEBPA* mutation	**15.2**	NR	**17.9**	3.1	20.4
*RUNX1* mutation	6.3	**15.2**	7.3	18.7	18.4
*ETV6* mutation	NR	3.7	NR	3.1	8.2
*GATA2* mutation	6.3	NR	2.8	1.9	2.0
*NPM1* mutation	**15.2**	11	11.2	**32.1**	16.3
*BCOR* mutation	NR	NR	NR	7.1	12.2
*ASXL1* mutation	**16.1**	**20.7**	**13.9**	13.2	6.1
*EZH2* mutation	NR	2.1	NR	3.6	8.2
*PHF6* mutation	2.7	4.8	NR	3.3	2.0
*U2AF1* mutation	2.7	4.3	NR	7.6	10.2
*SRSF2* mutation	NR	5.9	NR	22.7	6.1
*ZRSR2* mutation	NR	1.1	NR	NR	2.0
*STAG2* mutation	NR	NR	NR	5.4	10.2
*RAD21* mutation	NR	NR	NR	1.2	2.0
*SMC1A* mutation	NR	NR	NR	NR	2.0
*TP53* mutation	8.0	14.4	7.3	8.3	10.2

The underlined figures indicate the most common mutation found by each study, whereas the bold figures denote the three most common mutations that each study reported

*F*, female; *M*, male, *NR*, not reported

Furthermore, *TP53* mutation was an independent adverse factor for outcome in our study, which is comparable with other published results [5, 28, 32]. In a previous report, decitabine has been suggested as an effective treatment for AML patients with mutated *TP53* [5]. However, this observation is not supported by data from controlled clinical trials [4, 33]. New agents, such as the p53 reactivator APR-246 or the monoclonal anti-CD47 antibody magrolimab that have shown promising activity in this high-risk subset of AML, are currently in clinical development [34, 35]. Apart from the limited transplant access, the poor OS and RFS rates of our patients were possible because age > 65 years and unfavorable genetic mutations (especially the *TP53* mutation) were adverse factors. The reason why the old age in this study confers grave prognosis could be explained by less availability of HMAs utilization.

Overall, there are different genetic abnormalities in AML patients in each race. A comprehensive genetic investigation of AML and MDS patients could categorize patients’ risks and prognoses. Moreover, personalized treatment based on each molecular mutation in individual patients could improve their treatment responses and long-term survival outcomes.

The main limitation of this study was the low number of enrolled patients. Consequently, several genetic mutations could not reach a level of statistical difference for outcome measures between the wild-type and the mutated gene. Establishing the exact prevalence and clinical outcome of each mutation in Thai AML patients would be interesting but would require the collection and analysis of more cases.

## Conclusions

*FLT3*-ITD was the most common mutation in newly diagnosed Thai AML patients. *TP53* mutation and advanced age were independent, poor prognostic factors for patients’ survival. The genetic landscape of AML patients for each disease type, each age group, and each nation differ; hence, a comprehensive genetic investigation should guide the most suitable treatment to improve individual patients’ outcomes.

## Supplementary Information

ESM 1 (DOCX 21.5 kb).

## Data Availability

The datasets used and/or analyzed during the current study are available from the corresponding author on reasonable request.
