# Supplementary material for: Genetic alterations in Thai adult patients with acute myeloid leukemia and myelodysplastic syndrome—excess blasts detected by next-generation sequencing technique
Source: Ann Hematol. 2021 Apr 10;100(8):1983–93. doi: 10.1007/s00277-021-04513-z (PMC8285357; doi:10.1007/s00277-021-04513-z)
Supplement: Supplementary file 1 — (DOCX 21.5 kb). [file 277_2021_4513_MOESM1_ESM.docx]

**Table S1** Comparison of the molecular findings of the *de novo* AML group and the MDS combined with secondary AML

|  | ***De novo* AML group (n = 39)** | **MDS combined with secondary AML**  **(n = 10)** | ***P*-value** |
| --- | --- | --- | --- |
|  | **Positive results (%)** | **Positive results (%)** |  |
| *NPM1* mutation | 20.5 | 0 | 0.180 |
| *FLT3*-ITD mutation | 35.9 | 0 | ***0.044*** |
| *FLT3*-TKD mutation | 15.4 | 10 | 1.000 |
| *ANKRD26* mutation | 2.6 | 0 | 1.000 |
| *ASXL1* mutation | 0 | 30 | ***0.007*** |
| *ASXL2* mutation | 7.7 | 0 | 1.000 |
| *BCOR* mutation | 10.3 | 20 | 0.588 |
| *BRAF* mutation | 5.1 | 0 | 1.000 |
| *CBL* mutation | 5.1 | 10 | 0.504 |
| *CEBPA mutation* | *25.6* | *0* | 0.097 |
| *CSF3R* mutation | 2.6 | 0 | 1.000 |
| *DNMT3A* mutation | 23.1 | 30 | 0.690 |
| *EZH2* mutation | 7.7 | 10 | 1.000 |
| *ETV6* mutation | 2.6 | 30 | ***0.023*** |
| *GATA2* mutation | 2.6 | 0 | 1.000 |
| *IDH1* mutation | 2.6 | 0 | 1.000 |
| *IDH2* mutation | 5.1 | 0 | 1.000 |
| *JAK2* mutation | 2.6 | 0 | 1.000 |
| *KIT* mutation | 10.3 | 0 | 0.569 |
| *KDM6A* mutation | 2.6 | 0 | 1.000 |
| *KMT2A* mutation | 5.1 | 0 | 1.000 |
| *KRAS* mutation | 7.7 | 10 | 1.000 |
| *MYC* mutation | 2.6 | 0 | 1.000 |
| *MPL* mutation | 0 | 10 | 0.204 |
| *NF1* mutation | 5.1 | 10 | 0.504 |
| *NRAS* mutation | 7.7 | 30 | 0.090 |
| *PHF6* mutation | 2.6 | 0 | 1.000 |
| *PTPN11* mutation | 12.8 | 10 | 1.000 |
| *RAD21* mutation | 2.6 | 0 | 1.000 |
| *RUNX1* mutation | 12.8 | 40 | 0.070 |
| *STAG2* mutation | 5.1 | 30 | 0.051 |
| *SMC1A* mutation | 2.6 | 0 | 1.000 |
| *SRSF2* mutation | 0.0 | 30 | ***0.007*** |
| *TET2* mutation | 20.5 | 20 | 1.000 |
| *TP53* mutation | 7.7 | 20 | 0.267 |
| *U2AF1* mutation | 7.7 | 20 | 0.267 |
| *WT1* mutation | 25.6 | 10 | 0.419 |
| *ZBTB7A* mutation | 5.1 | 10 | 0.504 |
| *ZRSR2* mutation | 2.6 | 0 | 1.000 |

A *p*-value of < 0.05 indicates statistical significance.

*AML*, acute myeloid leukemia; *MDS*, myelodysplastic syndrome

**Table S2** Comparison of the molecular findings of the patients aged ≤ 65 years and > 65 years

|  | **Patients aged ≤ 65 years (n = 38)** | **Patients aged > 65 years (n = 11)** | ***P*-value** |
| --- | --- | --- | --- |
|  | **Positive results (%)** | **Positive results (%)** |  |
| *NPM1* mutation | 10.5 | 36.4 | 0.063 |
| *FLT3*-ITD mutation | 23.7 | 45.5 | 0.254 |
| *FLT3*-TKD mutation | 13.2 | 18.2 | 0.646 |
| *ANKRD26* mutation | 0.0 | 9.1 | 0.224 |
| *ASXL1* mutation | 5.3 | 9.1 | 0.542 |
| *ASXL2* mutation | 5.3 | 9.1 | 0.542 |
| *BCOR* mutation | 10.5 | 18.2 | 0.605 |
| *BRAF* mutation | 5.3 | 0.0 | 1.000 |
| *CBL* mutation | 2.6 | 18.2 | 0.122 |
| *CEBPA* mutation | 23.7 | 9.1 | 0.419 |
| *CSF3R* mutation | 2.6 | 0.0 | 1.000 |
| *DNMT3A* mutation | 26.3 | 18.2 | 0.708 |
| *EZH2* mutation | 7.9 | 9.1 | 1.000 |
| *ETV6* mutation | 5.3 | 18.2 | 0.214 |
| *GATA2* mutation | 2.6 | 0.0 | 1.000 |
| *IDH1* mutation | 0.0 | 9.1 | 0.224 |
| *IDH2* mutation | 2.6 | 9.1 | 0.402 |
| *JAK2* mutation | 0.0 | 9.1 | 0.224 |
| *KIT* mutation | 7.9 | 9.1 | 1.000 |
| *KDM6A* mutation | 0.0 | 9.1 | 0.224 |
| *KMT2A* mutation | 5.3 | 0.0 | 1.000 |
| *KRAS* mutation | 10.5 | 0.0 | 0.380 |
| *MYC* mutation | 2.6 | 0.0 | 1.000 |
| *MPL* mutation | 0.0 | 9.1 | 0.224 |
| *NF1* mutation | 7.9 | 0.0 | 1.000 |
| *NRAS* mutation | 10.5 | 18.2 | 0.605 |
| *PHF6* mutation | 2.6 | 0.0 | 1.000 |
| *PTPN11* mutation | 10.5 | 18.2 | 0.605 |
| *RAD21* mutation | 2.6 | 0.0 | 1.000 |
| *RUNX1* mutation | 15.8 | 27.3 | 0.400 |
| *STAG2* mutation | 7.9 | 18.2 | 0.311 |
| *SMC1A* mutation | 2.6 | 0.0 | 1.000 |
| *SRSF2* mutation | 2.6 | 18.2 | 0.122 |
| *TET2* mutation | 15.8 | 36.4 | 0.201 |
| *TP53* mutation | 10.5 | 9.1 | 1.000 |
| *U2AF1* mutation | 10.5 | 9.1 | 1.000 |
| *WT1* mutation | 23.7 | 18.2 | 1.000 |
| *ZBTB7A* mutation | 5.3 | 9.1 | 0.542 |
| *ZRSR2* mutation | 0.0 | 9.1 | 0.224 |

A *p*-value of < 0.05 indicates statistical significance

*AML*, acute myeloid leukemia; *MDS*, myelodysplastic syndrome
